# Do School Athletes Really Eat Better? Nutritional and Body Composition Differences in Saudi Adolescents

**DOI:** 10.3390/children13070852

**Published:** 2026-06-24

**Authors:** Ghareeb O. Alshuwaier

**Affiliations:** Department of Exercise Physiology, College of Sport Sciences and Physical Activity, King Saud University, Riyadh 11451, Saudi Arabia; galshuwaier@ksu.edu.sa

**Keywords:** physical inactivity, food frequency, overweight, central obesity, school sport, Arab adolescents, cross-sectional study, Saudi Vision 2030

## Abstract

**Background**: Obesity among Saudi adolescents has risen sharply, yet whether school athletic participation is associated with students showing improved dietary habits and better anthropometric profiles compared to those of their non-athlete peers remains unclear. This study compared anthropometric indices and dietary habits between school athletes and non-athletes in Riyadh. **Methods**: A cross-sectional study was conducted with 124 male secondary school students (70 athletes and 54 non-athletes aged 16–17 years) in Riyadh, Saudi Arabia. Athletes were defined as students who reported engaging in vigorous-intensity sport for ≥3 days/week for ≥60 min/session. BMI, body weight, and waist circumference were measured objectively. Dietary habit frequencies across ten food categories were assessed using the validated Arab Teens Lifestyle Study (ATLS) questionnaire. Independent samples t-tests and chi-square tests were used; effect sizes were calculated as Cohen’s d. A Bonferroni-corrected threshold (*p* < 0.005) was applied for multiple dietary comparisons. **Results**: Athletes had significantly lower BMI (23.64 ± 5.39 vs. 30.28 ± 7.25 kg/m^2^; *p* < 0.001, d = 1.06), body weight (*p* < 0.001, d = 0.93), and waist circumference (85.46 ± 12.61 vs. 95.50 ± 17.89 cm; *p* < 0.001, d = 0.66). Obesity prevalence was 15.7% among athletes versus 51.9% among non-athletes. Of ten dietary variables, only fresh fruit consumption showed a between-group difference (62.9% vs. 40.7% high-frequency; *p* = 0.010), which did not survive Bonferroni correction. **Conclusions**: School athletes demonstrated substantially better anthropometric profiles than their non-athlete peers, but dietary habit frequencies were largely similar across both groups. The high obesity prevalence among non-athletes underscores the need for school-based programs that combine structured physical activity with targeted nutrition education.

## 1. Introduction

Saudi Arabia has undergone a rapid and well-documented epidemiological transition over recent decades, characterized by accelerated urbanization, increasing sedentary behavior, and a pronounced dietary shift from traditional to Westernized food patterns [1,2]. The health consequences for the country’s young population have been severe. National surveillance data confirm that between 1988 and 2005, the prevalence of obesity among Saudi adolescents rose from 3.4% to 24.5%, and the upward trajectory has continued [3,4]. A recent multicenter population-based study across the National Guard Health System, encompassing five hospitals and 24 primary care centers, reported that approximately one-fifth of Saudi children and adolescents aged 2–19 years are overweight (11.2%) or obese (9.4%), with obesity prevalence being highest among boys (10.4%) and in the Central and Eastern regions of the kingdom [5]. These figures are particularly alarming given that obese children are substantially more likely to remain obese in adulthood and to develop non-communicable diseases including type 2 diabetes, cardiovascular disease, hypertension, and musculoskeletal disorders [6,7].

The obesogenic environmental facing Saudi youth is well characterized. A study identified sedentary lifestyles; unhealthy school canteen provision; inadequate physical education, particularly for females; and heavy reliance on motorized transport as key contributors to elevated obesity rates [4]. A study demonstrated that geographical location, neighborhood safety, access to recreation facilities, and cultural attitudes toward physical activity are all independently associated with body mass index (BMI) and physical activity (PA) levels among Saudi youth, with rural desert adolescents demonstrating both lower PA and higher BMI than their peers living in urban and rural farm areas [8]. Complementing this, Aljassim and Jradi identified paternal unemployment, paternal obesity, infrequent active play, excessive screen time, and incorrect parental perception of their child’s weight as independent risk factors for childhood overweight and obesity in Riyadh, findings that point to a multifactorial, environmentally embedded problem requiring a systemic solution [7].

The socioenvironmental determinants of adolescent adiposity in Saudi Arabia are well established and directly relevant to the present study. Socioeconomic status (SES) has been identified as a primary driver of both dietary quality and obesity: children from lower-income households in Riyadh were more likely to be overweight, while parental education and employment status independently predicted child weight status [7,9]. Pubertal and biological maturity status is an equally important confounder, given that the timing of the adolescent growth spurt substantially influences both BMI trajectories and PA engagement [10,11]. Additionally, parental BMI—particularly paternal BMI in the Saudi context, where fathers typically govern household activity decisions—has been shown to approximately double the likelihood of childhood obesity [7]. These variables are discussed in the context of the study’s limitations below.

Organized sport participation in school stinting has been proposed as one such systemic solution, offering structured physical activity opportunities that are equitable, supervised, and culturally accessible for male adolescents. A recent study demonstrated that non-athletic adolescents in Riyadh face significant health disadvantages characterized by a higher overweight and central obesity rate, and identified structural and social barriers, specifically the absence of suitable exercise facilities and exercise partners, as the primary impediments to PA engagement, rather than academic time pressure [12]. According to Self-Determination Theory, these barriers reflect unmet needs for competence and relatedness, suggesting that school-based physical education is a critical intervention setting. However, whether school athletics confers benefits specifically through dietary pathways, in addition to direct energy expenditure effects, remains underexplored in the Saudi adolescent population [12].

Dietary quality among Saudi adolescents is a significant independent concern. National dietary surveys indicate that Saudi youth consume excessive amounts of energy-dense, nutrient-poor food, with fat consumption per capita having risen to 143% of recommended levels, and widespread consumption of sugary drinks, fast food, and confectionery [4,13,14]. A study confirmed that while many Saudi adults are aware of the need to eat healthy food, three-quarters follow no specific dietary plan and adherence to structured dietary regimes is poor [15]. Studies involving primary school children across Tabuk [6] and Riyadh [7] have found that overweight and obese children have lower intake of breakfast, fruits, and dairy compared to their normal-weight peers, reinforcing the link between dietary patterns and weight status in Saudi youth. There is a widely held assumption that athlete populations demonstrate healthier dietary behaviors than their non-athletic peers, partly mediated by greater health awareness and partly due to the supportive dietary norms of sporting peer groups [16].

This study therefore aimed to determine whether school athletes demonstrate healthier dietary habit frequencies and better anthropometric profiles compared with their non-athlete peers in Riyadh secondary school. The study makes three specific contributions: (i) it provides a direct athlete/non-athlete comparison using validated regional instruments (ATLS) and IOTF-appropriate BMI criteria in Saudi secondary school males, a population that is under-represented in the international literature; (ii) it reports obesity prevalence in non-athletic adolescents, who are deemed to be a high-risk subgroup; and (iii) the null dietary findings themselves are informative, challenging the assumption that athletic participation promotes healthier eating in this population. The specific objectives were (1) to compare anthropometric profiles between athlete and non-athlete groups, including BMI categories, weight, and waist circumference; and (2) to compare dietary habit frequency patterns across ten food and beverage categories between the two groups.

## 2. Materials and Methods

### 2.1. Study Design and Participants

A cross-sectional comparative study was conducted in Riyadh, Saudi Arabia. Participants were recruited from four public secondary schools in Riyadh that had established sports programs. Schools were identified in collaboration with the relevant school administration, and eligible students were invited to participate during school hours. All students who provided written informed consent and met the inclusion criteria were enrolled. A total of 124 male high school students aged 16–17 years participated in the study. Participants were recruited from secondary schools in Riyadh. The sample was divided into two groups based on self-reported physical activity engagement: athletes (n = 70) and non-athletes (n = 54). Athletes were defined as students who self-reported engaging in vigorous-intensity sport or structured physical training on three or more days per week for a minimum of 60 min per session, a threshold consistent with established criteria for adolescent sport participation research [12]. This definition captures a broad, heterogeneous group of physically active adolescents and does not distinguish by sport type, competition level, or training history, a limitation that we acknowledge below. Participants who did not meet this threshold were classified as non-athletes. Ethical approval was obtained from the Institutional Review Board of King Saud University (Reference number: KSU-HE-22-891; approved 3 January 2023). All procedures were conducted in accordance with the Declaration of Helsinki. Written informed consent was obtained from all participants following a full explanation of the study’s purpose and procedures. Participants with chronic health conditions that could confound anthropometric or dietary measurements were excluded. A formal a priori power analysis (G*Power 3.1) using the observed effect size for BMI (d = 1.06) at α = 0.05 indicated that the achieved sample of 124 participants provides >99% power for the primary anthropometric comparisons and approximately 80% power to detect a medium effect (d = 0.50) for secondary outcomes.

### 2.2. Anthropometric Measurements

Standing height was measured to the nearest 0.1 cm using a calibrated stadiometer (Detecto Electronic, Model DHRWM, Webb City, MO, USA), with participants barefoot and with their backs straight. Body mass was recorded to the nearest 0.1 kg using a calibrated digital scale (Seca-869, Hamburg, Germany). Body mass index (BMI) was calculated as body mass (kg) divided by height squared (m^2^). Waist circumference (WC) was measured twice to the nearest 2 mm at the midpoint between the lower costal margin and iliac crest using a non-stretchable measuring tape (Seca 201, Hamburg, Germany), following procedures consistent with the work of Guilherme et al., 2015 [17]; the mean of the two measurements was used for analysis. The measurements were repeated if the two readings differed by more than 0.5 cm. All measurements were performed by two trained research assistants who had completed standardized anthropometric training; the measurements were taken during morning sessions between 08:00 and 11:00. The technical error of measurement (TEM) for waist circumference was ≤1.5%, consistent with acceptable intra-rater reliability standards for field-based anthropometric research. A waist-to-height ration threshold of 0.5 was considered the cut-off for elevated cardiometabolic risk, consistent with guidance published by McCarthy and Ashwell, 2006 [18].

BMI categories were assigned according to the International Obesity Task Force (IOTF) age- and sex-specific cut-off points, which have been validated for use in adolescent populations [17,19,20]: underweight (<5th percentile), normal weight (5th–84.9th percentile), overweight (85th–94.9th percentile), and obese (≥95th percentile). This approach is preferred over the application of adult World Health Organization (WHO) BMI threshold to adolescent populations, as it accounts for the age and sex-specific differences in anthropometric development [5,19].

### 2.3. Dietary Assessment

Dietary habit frequencies were assessed using the Arab Teens Lifestyle (ATLS) questionnaire, a validated instrument designed to evaluate health-related behaviors among adolescents and young adults aged 14–15 years [12,21]. The ATLS questionnaire has demonstrated good reliability and validity in previous studies and has been widely used in assessments of Saudi adolescent health behaviors [21]. It should be noted that the ATLS dietary module assesses consumption frequency only and does not capture portion sizes, energy intake, or macronutrient composition. Accordingly, the present study reports dietary habit frequencies rather than nutritional intake, and conclusions regarding diet quality or energy balance cannot be drawn from these data. Participants reported the weekly frequency of consumption of ten food and beverage items: breakfast, vegetables, fresh fruits, milk and dairy products, sugar-sweetened beverages, fast food, fried potatoes, donuts and cakes, sweet and chocolates, and energy drinks. Response options ranged from 0 (never) to 7 days per week. For categorical analysis, consumption frequencies were grouped as low (1–2 days/week), moderate (3–4 days/week), and high (≥5 days/week).

### 2.4. Statistical Analysis

Data were analyzed using SPSS statistical software (Version 28; IBM Crop, Armonk, NY, USA). The normality of continuous variables was assessed using the Shapiro–Wilk test; homogeneity of variance was verified using Levene’s test. Continuous anthropometric variables were compared between groups using an independent samples t-test, with effect sizes calculated as Cohen’s d (small ≥ 0.2, medium ≥ 0.5, large ≥ 0.8). Dietary habits variables were operationalized as categorical data and analyzed using Pearson’s chi-square tests of independence. To address the multiple comparisons problem arising from ten simultaneous dietary chi-square tests, a Bonferroni-corrected significance threshold was applied (α/10 = 0.005). All tests were two-tailed. An a priori power analysis (G*Power 3.1) using the observed between group effect size for BMI (d = 1.06) at α = 0.05 indicated that the achieved sample of 124 participants provided >99% power for the primary body composition and approximately 80% power to detect a medium effect (d = 0.50) for continuous secondary outcomes.

## 3. Results

### 3.1. Participants’ Characteristics

A total of 124 male secondary school students participated, comprising 70 athletes (56.5%) and 54 non-athletes (43.5%). Mean age was comparable between groups (athletes: 16.66 ± 0.63 years; non-athletes: 16.61 ± 0.63 years; t = 0.40, *p* = 0.688), confirming appropriate age matching.

### 3.2. Anthropometric Indices

Table 1 presents anthropometric comparisons between groups. Athletes had a significantly lower BMI (23.64 ± 5.39 vs. 30.28 ± 7.25 kg/m^2^; t = −5.85, *p* < 0.001, d = 1.06), body weight (72.56 ± 17.35 vs. 91.04 ± 22.71 kg; t = −5.14, *p* < 0.001, d = 0.93), and waist circumference (85.46 ± 12.61 vs. 95.50 ± 17.89 cm; t = −3.67, *p* < 0.001, d = 0.66) compared to non-athletes. No significant difference was observed for height (t = 1.43, *p* = 1.155, d = 0.26). Notable, the mean waist circumference of non-athletes (95.50 cm) exceeded the clinically relevant risk threshold (waist-to-height ratio > 0.5) when combined with their mean height, indicating elevated cardiometabolic risk in this group.

Table 2 present BMI category distributions. The overall distribution differed significantly between groups (χ^2^ = 26.71, df = 3, *p* < 0.001). More than half of athletes (55.7%) were classified as normal weight compared to only 18.5% of non-athletes. Conversely, obesity prevalence was substantially higher among non-athletes (51.9%) than athletes (15.7%). Underweight prevalence was 11.4 among athletes versus 3.7% among non-athletes, a clinically noteworthy finding that is discussed further below.

#### Dietary Habit Frequencies

Table 3 presents dietary habit frequency comparisons across ten food and beverage categories. Of the ten variables examined, only fresh fruit consumption differed significantly between groups (χ^2^ = 9.20, df = 2, *p* = 0.010); 62.9% of athletes were in the high-consumption category (≥5 days/week) compared to 40.7% of non-athletes, and fewer athletes were in the low-consumption category (10.0% vs. 29.6%). Applying the Bonferroni correction for ten simultaneous comparisons (adjusted α = 0.005), fresh fruit consumption did not survive correction (*p* = 0.010 > 0.005). Accordingly, this finding should be interpreted as exploratory and hypothesis-generating rather than confirmatory. No significant between-group differences were observed for breakfast consumption (*p* = 0.352), vegetable intake (*p* = 0.213), milk and dairy products (*p* = 0.631), sugar-sweetened beverages (*p* = 0.324), fast food (*p* = 0.349), or energy drinks (*p* = 0.854). The continuous analysis of dietary items also revealed a non-significant trend toward higher vegetable consumption in athletes (5.86 ± 2.17 vs. 5.15 ± 2.36 days/week; *p* = 0.085).

## 4. Discussion

This study investigates whether school athletes demonstrate superior anthropometric profiles and healthier dietary habit frequencies compared to their non-athlete peers in secondary schools in Riyadh. This study contributes to the literature in three ways: (1) it provides the first direct comparison using the validated ATLS instrument and IOTF-appropriate BMI criteria in Saudi secondary school males; (2) the 51.9% obesity prevalence reported in the non-athlete group identifies this subgroup as carrying a disproportionate cardiometabolic risk burden; and (3) the null dietary findings challenge the assumption that athletic participation promotes broader dietary change in this population. The principal findings are as follows: athletes showed markedly better anthropometric outcomes, with large effect sizes for BMI and weight differences; and dietary habit frequencies were largely similar between groups, with only a borderline and non-significant difference in fresh fruit consumption.

The anthropometric differences between groups were clinically substantial. The obesity prevalence of 51.9% among non-athletes is among the highest reported in any single study on Saudi adolescent males, and aligns with—and exceeds—the broader national picture of escalating overweight and obesity trends documented across multiple studies [3,5,6]. A study identified using the Saudi growth chart across a national cohort of 337,316 Saudi children and adolescents, reported overall obesity overweight prevalence of 9.4% and 11.2%, respectively, substantially lower than those found in our non-athletes group. This discrepancy likely reflects the highly selected and sedentary nature of the non-athlete group in the present study, rather than a population-level estimate, and underlines that non-athletic adolescents represent a particularly high-risk subgroup within the broader Saudi adolescent population. The very large effect size for BMI differences (Cohen’s d = 1.06) was consistent with findings from a recent study in a comparable Riyadh sample [12], strengthening confidence in the robustness of these associations. However, the cross-sectional design does not permit causal inference: reverse causation, whereby leaner adolescents are more likely to self-select into sport, was equally plausible and cannot be excluded.

The waist circumference findings carry particular clinical significance. The mean waist circumference of non-athletes (95.50 cm) substantially exceeded the risk threshold associated with cardiometabolic risk. A study demonstrated, in a principal component analysis of CVD risk factors in school-aged children, that measures of adiposity, including waist circumference and BMI, loaded most highly on the dominant risk component, explaining 34% of variance in cardiovascular risk indicators and reinforcing that abdominal adiposity was among the most important modifiable risk factors in the young population [19]. The waist circumference differences observed in the present study (approximately 10 cm between groups) therefore represent clinically meaningful, and potentially preventable, cardiometabolic risk associated with non-athletic status. A recent study further demonstrated that neighborhood-level environmental factors, including lack of recreational facilities and perceived neighborhood unsafety, independently predict higher BMI and lower PA in Saudi youth [8], suggesting that the poor anthropometric profile of the non-athlete group reflects not only individual behaviors but also the broader built environment.

A noteworthy finding was the 11.4% underweight prevalence among athletes. This has not been discussed in the context of existing Saudi literature but warrants clinical attention. Relative Energy Deficiency in Sport (RED-S), a syndrome arising from insufficient energy availability relative to training demands, has been increasingly well documented in adolescent athletic populations globally, and is known to affect male athletes. The combination of high physical training loads and potentially inadequate dietary intake among some athletes in this study may place a subset at risk of RED-S. Future research should include measures of dietary energy intake and expenditure alongside anthropometric assessments to explore this possibility.

The near-uniform dietary habit frequency patterns across both groups are a significant public health finding. The absence of meaningful differences between athletes and non-athletes, particularly regarding sugar-sweetened beverages and fast food, suggests that athletic participation alone does not translate into broadly healthier eating behaviors in this Saudi adolescent population [15]. This was consistent with the findings of Alhusseini et al. [15], who reported that the majority of Saudi adults do not follow structured dietary plans irrespective of exercise behavior. A study attributed this partly to the shift from traditional Saudi food culture toward Westernized fast food consumption, a transition that has affected Saudi youth broadly and appears to operate independently of athletic status [4]. Aljassim and Jradi, 2021 [7], reinforced this, showing that fast food and sugary drink consumption did not significantly differ between overweight/obese and normal-weight children in a Riyadh case–control study. The borderline fresh fruit difference (*p* = 0.010, non-significant after Bonferroni correction) represents an exploratory signal that may warrant future investigation in adequately powered longitudinal designs, where potential confounders such as SES can be controlled [7].

These findings have direct implications for school-based health intervention design in Saudi Arabia. Given that the association between athletic participation and better anthropometric profiles appears to be driven primarily by energy expenditure rather than dietary modification, comprehensive interventions are required that address both domains simultaneously. A study argued that effective childhood obesity prevention requires multi-agency collaboration across schools, health services, community leaders, and the food industry [4]. Al-Nuaim et al. specifically recommended investment in accessible indoor recreational facilities to address the climate and safety barriers that disproportionately restrict Saudi adolescents’ physical activity [8]. School-based PA promotion combined with embedded nutrition education represents the most evidence-aligned approach for this population [12].

Several limitations of this study require acknowledgment. First, the cross-sectional design precludes causal inferences about whether athletic participation reduces obesity risk or whether leaner students self-select into sport teams [22]. Second, pubertal and biological maturity status was not assessed. The timing of the adolescent growth spurt substantially influences both BMI and PA trajectories, and its absence as a covariate is a meaningful limitation [11]. Third, the exclusively male sample limits generalizability to female adolescents, who face distinct barriers to physical activity in Saudi Arabia [4,8]. Fourth, socioeconomic status was not captured, despite being a primary determinant of both dietary quality and obesity in Saudi children [7]. Fifth, parental BMI and educational attainment, established independent predictors of childhood overweight in the Saudi context [7,8], were not assessed. Sixth, the dietary assessment used weekly frequency data without portion sizes, precluding energy intake quantification. Seventh, the athlete classification was based on self-reported vigorous PA frequency and does not distinguish by sport type, competition level, or training volume, limiting the characterization of athletic exposure. Eighth, as noted, the Bonferroni-corrected analysis rendered the fresh fruit finding non-significant; future adequately powered studies with pre-specified dietary hypotheses are needed to confirm this result.

## 5. Conclusions

School athletes demonstrated markedly better anthropometric profiles than their non-athlete peers (obesity: 15.7% vs. 51.9%; BMI Cohen’s d = 1.06). While dietary habit frequencies were largely similar, only fresh fruit consumption showed a directional difference favoring athletes, and this did not survive Bonferroni correction. These findings are associational: the cross-sectional design does not permit causal inference, and reverse causation cannot be excluded. These findings call for school-based interventions that embed structured PA for all adolescents alongside evidence-based nutrition education. Future research should include female participants, longitudinal designs, and measures of dietary energy intake, socioeconomic status, pubertal maturity, and neighborhood environment.

## Figures and Tables

**Table 1:** Comparison of anthropometric variables between athletes and non-athletes.

Variable	Athletes (n = 70) Mean ± SD	Non-Athletes (n = 54) Mean ± SD	t	*p*	Cohen’s d
Age (years)	16.66 ± 0.63	16.61 ± 0.63	0.40	0.688	0.07
Height (m)	1.75 ± 0.05	1.73 ± 0.07	1.43	0.155	0.26
Weight (kg)	72.56 ± 17.35	91.04 ± 22.71	−5.14	<0.001 ***	0.93
BMI (kg/m^2^)	23.64 ± 5.39	30.28 ± 7.25	−5.85	<0.001 ***	1.06
Waist (cm)	85.46 ± 12.61	95.50 ± 17.89	−3.67	<0.001 ***	0.66

Note. Data presented as mean ± SD. *** *p* < 0.001.

**Table 2:** Distribution of BMI categories among athletes and non-athletes.

BMI Category	Athletes (n = 70) n (%)	Non-Athletes (n = 54) n (%)
Underweight	8 (11.4)	2 (3.7)
Normal weight	39 (55.7)	10 (18.5)
Overweight	12 (17.1)	14 (25.9)
Obese	11 (15.7)	28 (51.9)
χ^2^ (df = 3)		26.71, *p* < 0.001 ***

Note: BMI categories based on IOTF age- and sex-specific cut-offs. *** *p* < 0.001.

**Table 3:** Comparison of dietary habit frequencies between athletes and non-athletes.

Dietary Item	Freq.	Athletes n (%)	Freq.	Non-Athletes n (%)	χ^2^	*p*
Breakfast	Low	12 (17.1)	Low	9 (16.7)	2.09	0.352
	Mod	19 (27.1)	Mod	9 (16.7)		
	High	39 (55.7)	High	36 (66.7)		
Sugary drinks	Low	20 (28.6)	Low	13 (24.1)	2.25	0.324
	Mod	27 (38.6)	Mod	28 (51.9)		
	High	23 (32.9)	High	13 (24.1)		
Vegetables	Low	6 (8.6)	Low	10 (18.5)	3.09	0.213
	Mod	14 (20.0)	Mod	12 (22.2)		
	High	50 (71.4)	High	32 (59.3)		
Fresh fruits ^†^	Low	7 (10.0)	Low	16 (29.6)	9.20	0.010 *
	Mod	19 (27.1)	Mod	16 (29.6)		
	High	44 (62.9)	High	22 (40.7)		
Milk/dairy	Low	6 (8.6)	Low	7 (13.0)	0.92	0.631
	Mod	20 (28.6)	Mod	17 (31.5)		
	High	44 (62.9)	High	30 (55.6)		
Fast food	Low	18 (25.7)	Low	20 (37.0)	2.10	0.349
	Mod	32 (45.7)	Mod	19 (35.2)		
	High	20 (28.6)	High	15 (27.8)		
Energy drinks	Low	57 (81.4)	Low	46 (85.2)	0.32	0.854
	Mod	10 (14.3)	Mod	6 (11.1)		
	High	3 (4.3)	High	2 (3.7)		

Note: Low = 1–2 days/week; mod = 3–4 days/week; high = ≥5 days/week. * *p* < 0.05 (does not survive Bonferroni correction; adjusted threshold *p* < 0.005). † Exploratory finding only.

## Data Availability

The data are available from the corresponding author upon reasonable request.
